# Supplementary material for: Engineering tumor-colonizing E. coli Nissle 1917 for detection and treatment of colorectal neoplasia
Source: Nat Commun. 2024 Jan 20;15:646. doi: 10.1038/s41467-024-44776-4 (PMC10799955; doi:10.1038/s41467-024-44776-4)
Supplement: Supplementary file 1 — Supplementary Information [file 41467_2024_44776_MOESM1_ESM.pdf]

Supplementary Information for

Engineering tumor-colonizing *E.coli* Nissle 1917 for detection and treatment of colorectal neoplasia

Candice R. Gurbatri†, Georgette A. Radford†, Laura Vrbanac, Jongwon Im, Elaine M. Thomas, Courtney Coker, Samuel R. Taylor, YoungUk Jang, Ayelet Sivan, Kyu Rhee, Anas A. Saleh, Tiffany Chien, Fereshteh Zandkarimi, Ioana Lia, Tamsin RM Lannagan, Tongtong Wang, Josephine A. Wright, Hiroki Kobayashi, Jia Q. Ng, Matt Lawrence, Tarik Sammour, Michelle Thomas, Mark Lewis, Lito Papanicolas, Joanne Perry, Tracy Fitzsimmons, Patricia Kaazan, Amanda Lim, Alexandra M Stavropoulos, Dion A. Gouskos, Julie Marker, Cheri Ostroff, Geraint Rogers, Nicholas Arpaia, Daniel L Worthley, Susan L Woods*, and Tal Danino*

*Correspondence should be addressed to T.D. (td2506@columbia.edu) and S.L.W. (susan.woods@adelaide.edu.au)

This PDF file includes:

List of Supplementary Materials

Supplementary Figure 1. EcN can colonize and release payloads within adenomas of varying stage
Supplementary Figure 2. Establishment of orthotopic CRC transplant mouse model
Supplementary Figure 3. Staining for the presence of EcN in CRC transplant orthotopic mouse model
Supplementary Figure 4. Visualizing gram negative bacteria in human CRC
Supplementary Figure 5. Strain specific EcN PCR detection assay: Primer design and culture enrichment for microbial sequences
Supplementary Figure 6. Enrichment of EcN in stool samples from neoplasia-bearing animals over normal controls after oral dosing
Supplementary Figure 7. Effect of salicylate on EcN growth kinetics
Supplementary Figure 8. Orally-delivered EcN producing PD-L1 and CTLA-4 blocking nanobodies and GM-CSF reduces tumor burden in ApcMin/+ mice
Supplementary Figure 9. Metabolite detection in distinct APCMin/+ cohorts
Supplementary Figure 10. Automated analysis of immunohistochemical staining
Supplementary Table 1. Clinical characteristics of Mutaflor CRC trial cohort

Supplementary Note. Clinical trial study protocol

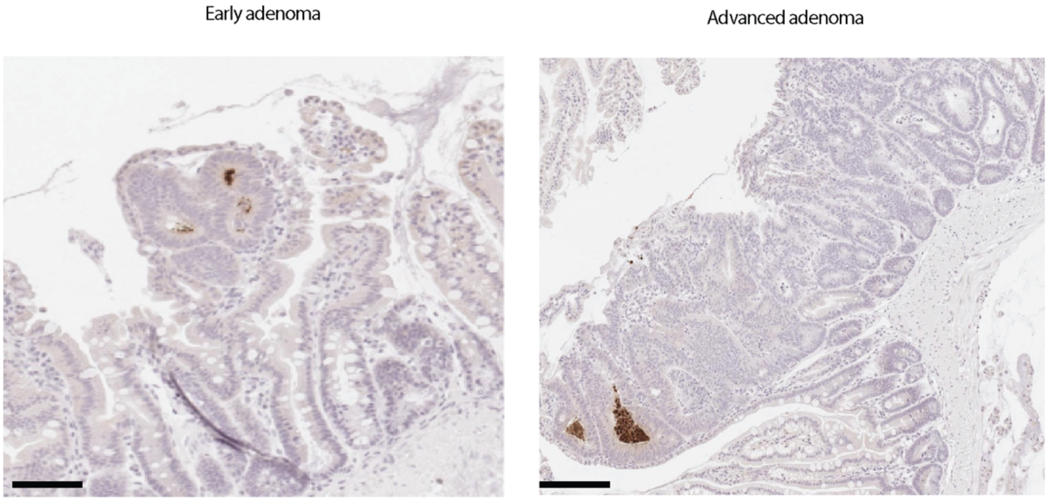

Supplementary Figure 1. EcN can colonize and release payloads within adenomas of varying stage. Apc$^{Min/+}$ mice were gavaged twice, 3-4 days apart, with EcN producing an HA-tagged reporter protein to enable protein detection in intestinal tissue by anti-HA immunohistochemistry after sacrifice at 4 weeks post-dosing (n=3 mice). Dark brown stain depicts HA-tagged protein in early and late-stage adenomas. Data corresponds to Fig. 1E. Error bars are 200μm.

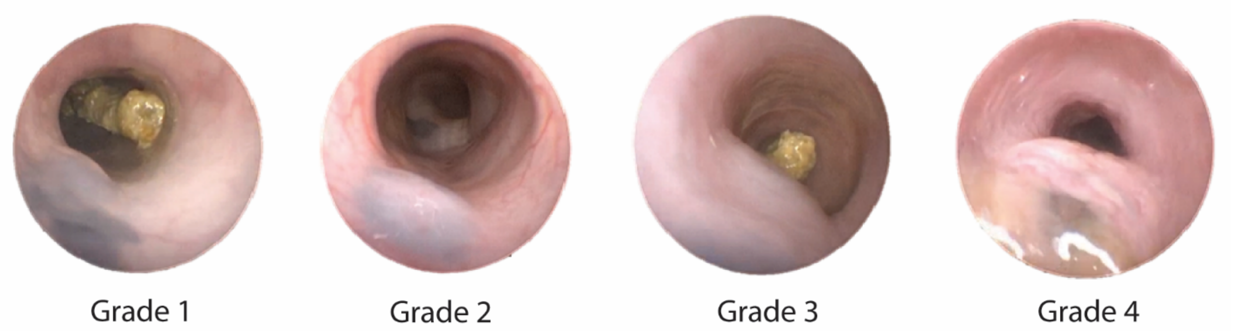

Supplementary Figure 2. Establishment of orthotopic CRC transplant mouse model. Colonoscopic images of mouse tumors growing into the distal colon lumen, grade 1 (smallest) to grade 4 (largest), as previously described[1]. Mice were treated once tumors had reached grades 3-4, for experiments depicted in Fig 2.

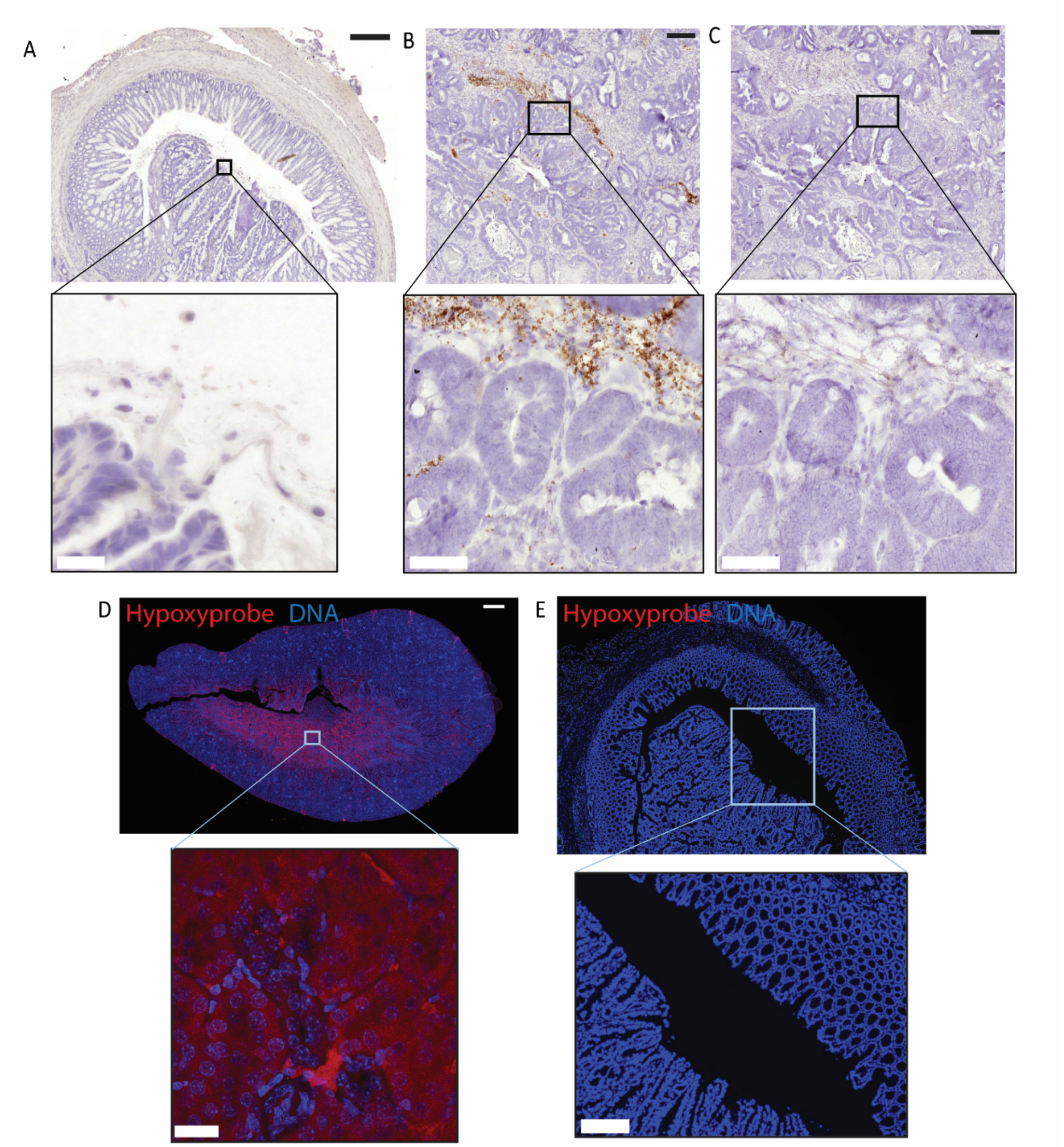

Supplementary Figure 3. Staining for the presence of EcN in CRC transplant orthotopic mouse model. (A) Experimental schema as depicted in Fig. 2A.Orthotopic CRC tumor from mouse orally dosed twice, 2 days apart, with EcN-lux. Representative RNA ISH staining for negative control probe, *DapB* (brown) on adjacent section to Figure2F (n = 5). Scale bar: 250μm & 25μm (lower inset). **(B-C)** Mouse CRC tissue intratumorally injected with EcN-lux bacteria after harvest, to generate a positive control tissue containing bacteria. Representative RNAscope ISH staining for (**B)** EcN-lux (brown) and (**C)** negative control probe, *DapB* (brown) (n = 2). Scale bar: 200μm & 50μm (lower inset). (**D)** Representative IF staining of positive control tissue, kidney medulla, for Hypoxyprobe (red, n = 17). Scale bar: 500μm & 20μm (lower inset). (**E)** Representative IF staining for Hypoxyprobe of a negative control orthotopic CRC tumor from a mouse that was injected with PBS rather than Hypoxyprobe agent prior to harvest (n = 4). Scale bar: 200μm.

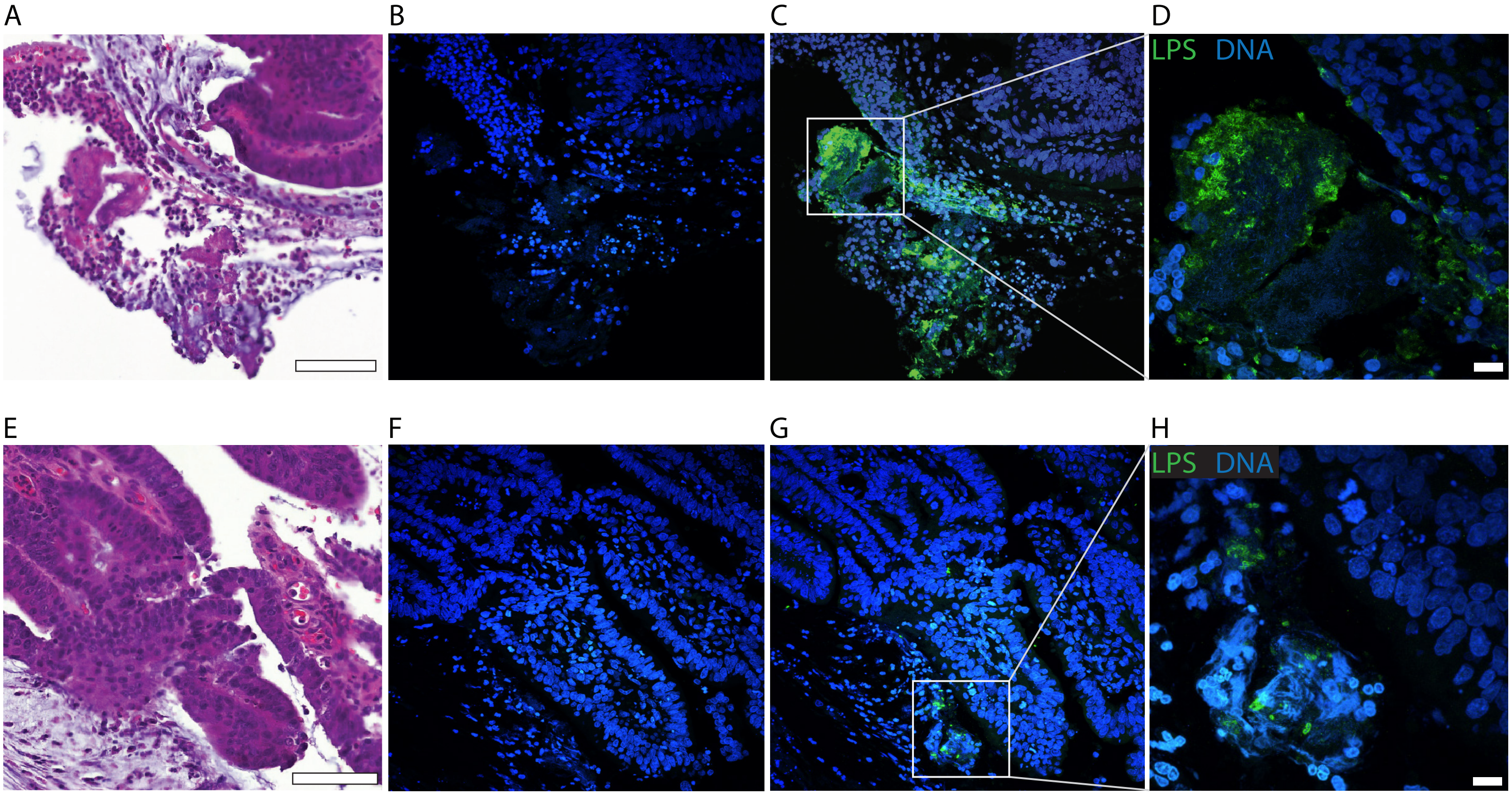

Supplementary Figure 4. Visualizing gram negative bacteria in human CRC. (A) Representative images from histopathological analysis of tumor tissue resected from CRC patients (n = 7 patients). Serial sections of region of interest one (A-D) and two (E-H), stained with (A, E) H&E, immunofluorescent stain with no primary control (B,F) or against lipopolysaccharide (LPS, green, C, D, G, H). Scale bars A, E 100μm, D, H 10μm .

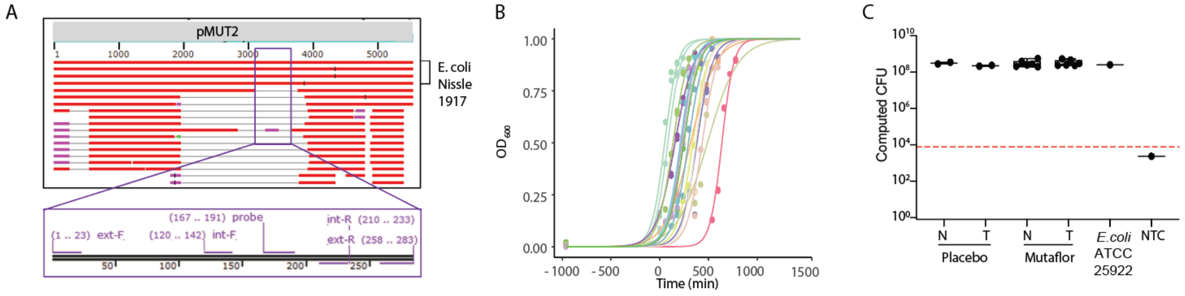

Supplementary Figure 5. Strain specific EcN PCR detection assay: Primer design and culture enrichment for microbial sequences. Redesigning a strain-specific EcN PCR assay to avoid potential false positive detection of other gut microbes in human tissue samples. **(A)** Full length endogenous pMUT2 plasmid sequence from EcN was used to BLAST against human gut microbiota DNA sequences to identify regions specific to EcN[2]. Red bars indicate regions with a BLAST alignment score of >200, magenta indicates a score of 80-200, green indicates a score of 50-80, grey line indicates non-homologous sections. DNA region boxed in purple contains 283bp DNA specific to EcN pMUT2 (3372-2654bp) and not other closely related sequences. PCR primers and probe were designed to this region as indicated. **(B-C)** Matched normal and tumor tissue homogenates from CRC patients administered placebo (n = 2) or Mutaflor (trade name for EcN) (n = 6) for 2 weeks. **(B)** Tissue samples were used to inoculate liquid culture for microbial enrichment. Microbial growth dynamics as measured by OD_{600} over 24h in batch liquid culture from n=8 patients, normal and tumor tissue homogenates in normoxia at 37 degrees C. **(C)** DNA isolated from culture was subjected to pan-microbial 16S, together with EcN specific assay depicted in Fig 2K. Mean value of 4 technical replicates shown per sample, bar at median per group. Red dashed line depicts the lower limit of detection of the assay based on standard curve dilution series, dot points above the line have detectable PCR amplicon signal. No signal was detected in no template (NTC), or buffer only DNA prep controls.

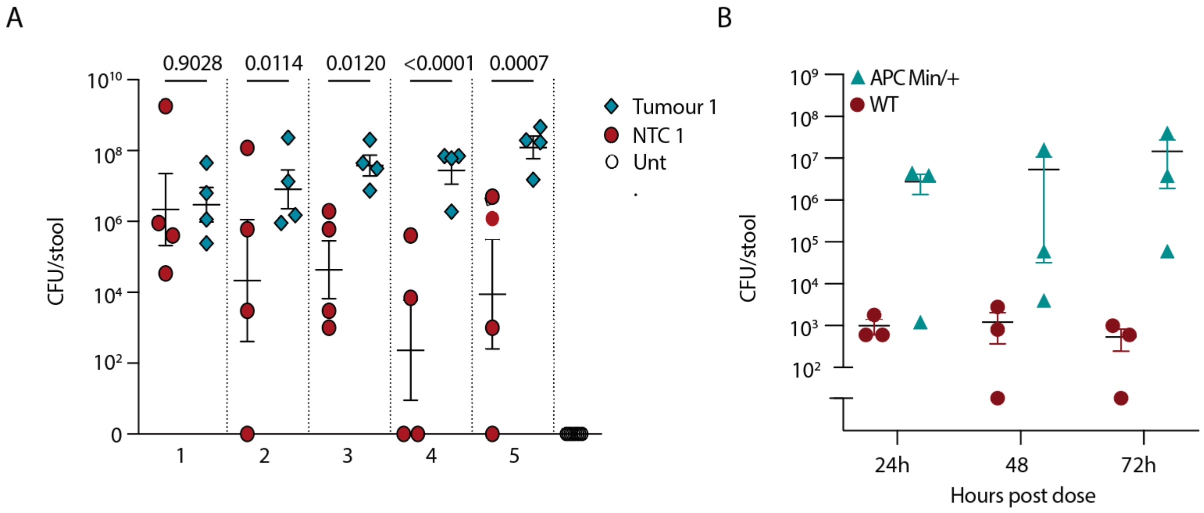

Supplementary Figure 6. Enrichment of EcN in stool samples from neoplasia-bearing animals over normal controls after oral dosing. (A) From the MSI CRC model, stool was collected from day 0 to 5 after the final EcN dose and plated on antibiotic-selective plates. (No tumor controls n = 4 - 5 stools, tumor bearing mice n = 4 stools, tumor-bearing PBS controls n = 9). * p < 0.05, *** p < 0.0005, **** p < 0.0001, two-way ANOVA, Fischer's LSD test). **(B)** 12-week-old *Apc*$^{Min/+}$ mice were gavaged twice, 3-4 days apart with 10^9 CFU bioluminescent EcN*ΔclbA*. Stool was collected 24, 48, and 72h after last dose, homogenized, plated on antibiotic-selective plates and quantified for CFU (n=3, n=1 stool per mouse). Source data are provided as a Source Data File.

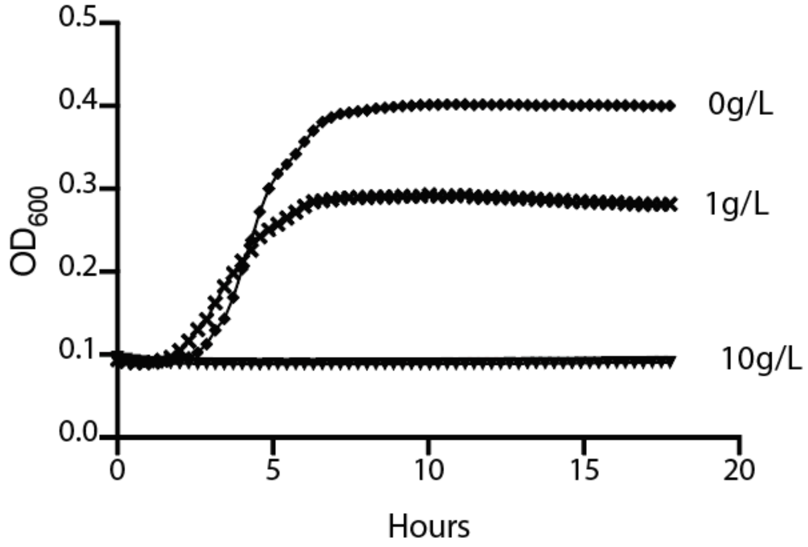

Supplementary Figure 7. Effect of salicylate on EcN growth kinetics. Plate reader data of EcN growth kinetics where an OD_{600} of 0.01 EcN was seeded on a 96 well plate and either 0, 1, or 10 g/L of salicylate solution was added to the wells. Data is of 3 technical replicates with mean +/- SEM shown for each condition. Source data are provided as a Source Data File.

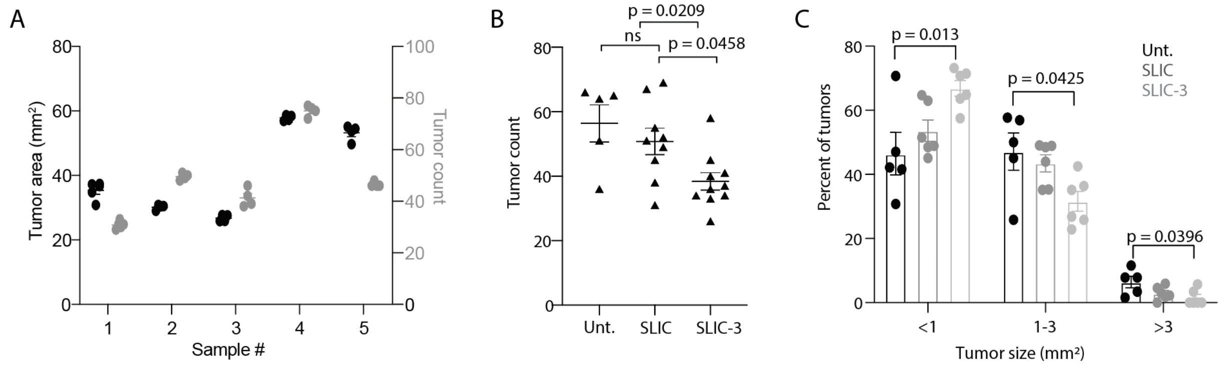

Supplementary Figure 8. Orally-delivered EcN producing PD-L1 and CTLA-4 blocking nanobodies and GM-CSF reduces tumor burden in *Apc^{Min/+}* mice. 15–17-week-old *Apc^{Min/+}* mice were dosed with PBS (Unt), EcN genomically encoding a lysis circuit (SLIC) or SLIC producing granulocyte-macrophage colony-stimulating factor (GM-CSF), and blocking nanobodies against PD-L1 and CTLA-4 targets (SLIC-3). 1 month after dosing, mice were sacrificed, intestines were bisected, swiss-rolled, paraffin embedded, sectioned, stained with hemotoxylin and eosin. Multiple sections (n=5 sections for sample 1 and 4 sections each for sample 2-5) in the same sample (n=5 samples) were quantified for **(A)** tumor area and tumor count to ensure that a single section was indeed representative of tumor metrics, (**B**) total tumor count based on a single section (n =5 mice in the untreated group, n= 9 mice in SLIC group, n= 10 mice in SLIC-3 group, one-way ANOVA with Holm-Sidak post test, error bars represent mean +/- SEM) and **(C)** percent of tumors <1 mm^2, 1-3 mm^2, or <3 mm^2 (n = 5 mice in the untreated group, n= 6 mice in SLIC group, n= 6 mice in SLIC-3 group , one-way ANOVA with Holm-Sidak post test, error bars represent mean +/- SEM). Corresponds to data shown in Fig. 4B. Source data are provided as a Source Data File.

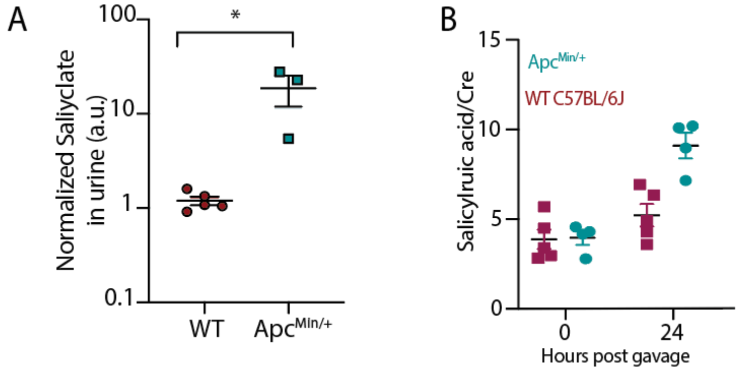

Supplementary Figure 9. Metabolite detection in distinct APCMin/+ cohorts. In two separate cohorts, 15-week-old APC$^{Min/+}$ mice were dosed with 10^9 EcN-SA bacteria and urine was collected 24hr after dosing. (A) LC-MS quantification of salicylate and (B) salicyuric molecules in urine of wild-type (WT) and APC$^{Min/+}$ mice (* $p = 0.015$, unpaired T test, n = 5 WT mice and n = 3 APC$^{Min/+}$ mice). Source data are provided as a Source Data File.

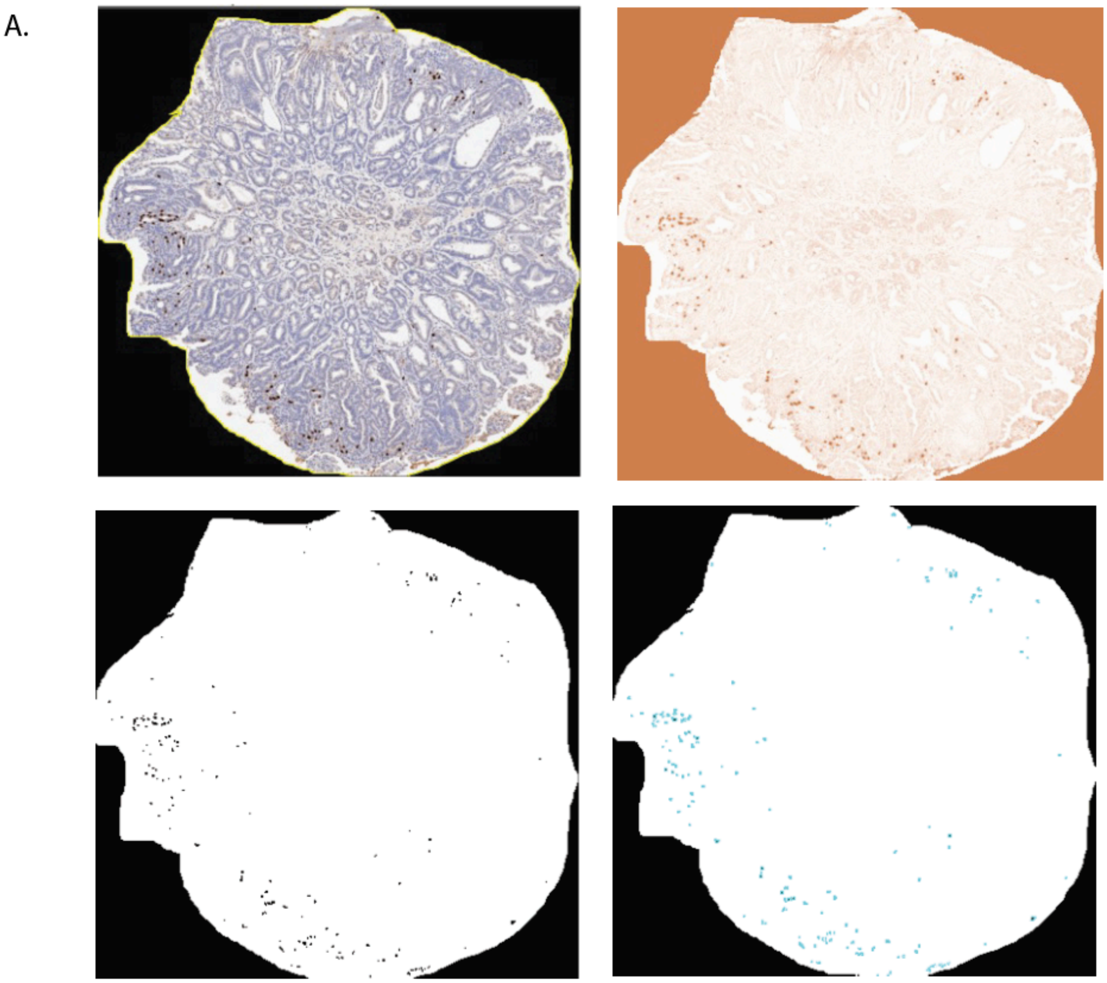

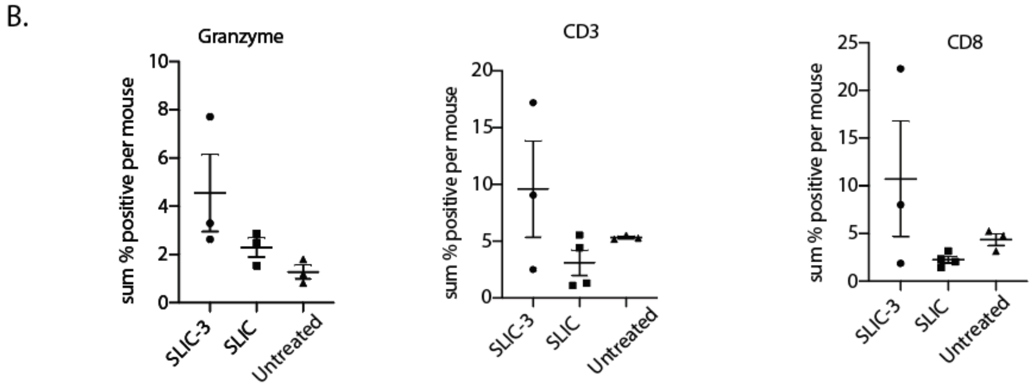

Supplementary Figure 10. Automated analysis of immunohistochemical staining. (A) Automated analysis of IHC staining where images represent (top left) manual segmentation of adenoma, (top right) devoncoluted image, (bottom left) thresholded image, (bottom right) measuring of the foreground pixels. (B) Related to Fig. 4E-G, the total % positive count per mouse (Granzyme, n = 3 mice per group, CD3 and CD8, n = 3

SLIC-3 mice, n = 4 SLIC mice, n = 3 Untreated mice) . Source data are provided as a Source Data File.

Supplementary Table 1. Clinical Characteristics of CRC trial cohort

Sex	Treatment group	CRC Stage	Location of primary CRC and adjacent normal tissue sampled
F	Mutaflor	pT1N1a	Transverse
M	Placebo	pT1N1M0	Rectosigmoid
M	Placebo	pT4aN3M1	Ascending
M	Mutaflor	pT3N0M0	Ascending
M	Mutaflor	pT3N0	Ascending
F	Mutaflor	pT2N1aM0	Rectal
F	Mutaflor	pT1N0M0	Ascending
M	Mutaflor	pT3N0M0	Ascending

References

1. C. Becker, M. Fantini, M. Neurath, High resolution colonoscopy in live mice. Nature protocols 1, 2900-2904 (2006).
2. A. Almeida, A. Mitchell, M.Boland, S. Forster, G. Gloor, A. Tarkowska, T. Lawley & R. Finn. Nature 568: 499-504 (2019).

Supplementary Note. Clinical trial study protocol

RESEARCH PROTOCOL

Full Title: *Colonisation of Probiotic in Neoplasia*

STUDY INVESTIGATOR(S)

Principal Investigator(s):

Dr Daniel Worthley M.B., B.S.(Hons), PhD, MPH, FRACP<br><br>Affiliations: SAHMRI<br><br>Position: Head Gastrointestinal cancer biology group<br><br>Responsibilities: Co-ordinating project, participant recruitment, obtaining consent, sample collection, data collection and analysis	

Sub- Investigator(s):

Dr Michelle Thomas M.B., B.S., FRACP<br><br>Affiliations: Royal Adelaide Hospital<br><br>Position: Unit Head, Staff Specialist Colorectal Surgeon<br><br>Responsibilities: Participant recruitment, obtaining consent, sample collection	Dr Matthew Lawrence M.B., B.S., FRACP<br><br>Affiliations: Royal Adelaide Hospital<br><br>Position: Staff Specialist Colorectal Surgeon<br><br>Responsibilities: Participant recruitment, obtaining consent, sample collection
A/Professor Tarik Sammour M.B., B.S., PhD, FRACP<br><br>Affiliations: Royal Adelaide Hospital and University of Adelaide<br><br>Position: Assoc Prof in Colorectal Surgery<br><br>Responsibilities: Participant recruitment, obtaining consent, sample collection	Dr Susan Woods, PhD<br><br>Affiliations: University of Adelaide<br><br>Position: Senior Scientist, Gastrointestinal cancer biology group<br><br>Responsibilities: Participant recruitment, obtaining consent, sample collection, data collection and analysis
Dr. Amanda Lim, M.B., B.S.,<br><br>Affiliations: Royal Adelaide Hospital<br><br>Position: Registrar, Dept of Gastroenterology & Hepatology,<br><br>Responsibilities: Accessing clinical information of participants, data collection.	Dr. Patricia Kaazan, M.B., B.S.,<br><br>Affiliations: Royal Adelaide Hospital<br><br>Position: Registrar, Dept of Gastroenterology & Hepatology,<br><br>Responsibilities: Accessing clinical information of participants, data collection.
Dr Josephine Wright, PhD<br><br>Affiliations: University of Adelaide	Dr Laura Vrbanac, PhD<br><br>Affiliations: University of Adelaide

Position: Research Fellow, Gastrointestinal cancer biology group<br>Responsibilities: Participant recruitment, obtaining consent, sample collection, data collection and analysis	Position: Research Fellow, Gastrointestinal Cancer Biology group<br>Responsibilities: Participant recruitment, obtaining consent, sample collection, data collection and analysis

1. INTRODUCTION

This is an interventional, double-blind, dual-centre, prospective clinical trial to evaluate the colonisation of matched human normal and neoplastic bowel tissue by the probiotic *E. coli* Nissle 1917 (EcN). Adult participants undergoing routine colonoscopy or surgical resection for primary colorectal cancer will be recruited from St. Andrew's Hospital and the Royal Adelaide Hospital, Adelaide (N=35). Written, informed consent will be provided before participants are assigned to take either 2 tablets (10^9 CFU) per day of non-genetically modified EcN (Mutaflor) or placebo for 14 days, prior to their procedure. Patients and treating physicians will be blind to active or placebo status. Mucosal biopsies (colonoscopy) or surgical resection samples from normal and neoplastic tissue will be collected from each participant at the time of their procedure. Participants will be excluded if they took probiotics or antibiotics during the trial period. If we can show that the probiotic EcN is enriched within neoplastic tissue in patients, as it is in mouse models of cancer, this will establish the foundation for future translational research using this probiotic for the detection and prevention of cancer.

2. BACKGROUND

Colorectal cancer (CRC, also known as bowel cancer) is common and costly. Our current approach to care is to use fecal and colonoscopic screening to try and identify, and then treat, early stages of disease. We need more economical and acceptable modes of CRC screening, prevention and therapy. The colonic microbiome is perfectly situated to serve as a bowel cancer bio-detector and, perhaps, even as a bio-preventative or therapy.

Several studies including our own have demonstrated that the administration of probiotics, specifically *Escherichia coli Nissle* 1917 (EcN), selectively colonizes colorectal tumors at both the metastatic and primary sites (Fig 1)[1]. But, does this also occur in humans? We don't need to use an engineered probiotic to test this. We simply need to measure whether an unmodified, commercially available probiotic colonises human tumors as it does in mice. The mechanism of action for EcN colonising polyps and metastases is currently unknown. Possible mechanisms include an immune suppressed local microenvironment in the tumour that allows bacterial growth, release of bacterial attractants from the necrotic core of the lesion or preferential growth of the bacteria in a hypoxic tumour environment.

To this end, we propose a short-term, interventional, double-blind, dual-centre, prospective clinical trial to ask the question: does EcN preferentially colonise colorectal polyps, tumors and even metastatic disease? EcN is a commonly used probiotic with a history of use in people for over a century, it is readily available in Australia, it has been used as a therapy in patients[2,3], and our colorectal surgical unit at the Royal Adelaide Hospital (RAH) has experience studying probiotics to reduce surgical complications.

We aim to recruit adult patients diagnosed with primary colorectal cancer scheduled for surgical resection or patients with precancerous lesions undergoing routine colonoscopy. Patients will take 10^9 colony forming units (CFU) per day of EcN (Mutaflor) or placebo for 14 days, prior to tissue resection via surgery or colonoscopy. This has been shown to be safe in earlier phase I trials that unfortunately did not measure tissue colonisation as an end-point[2]. We also plan to recruit patients scheduled for hepatic resection of colorectal cancer metastasis to examine colonization of advanced disease. A 9ml blood sample will be collected from each participant at the time of their procedure. Pathologically surplus tissue from the neoplasia and normal mucosa will be used to test whether more EcN probiotic is present in the neoplastic tissue than normal colonic mucosa, and in metastatic disease but not normal resected liver. We will use high sensitivity, PCR-based assays to also screen blood samples for the presence of EcN in blood. Understanding whether neoplastic specific homing and colonization of EcN occurs in humans, as it does in mouse models, will be an important step towards the use of probiotic strains for cancer detection and treatment.

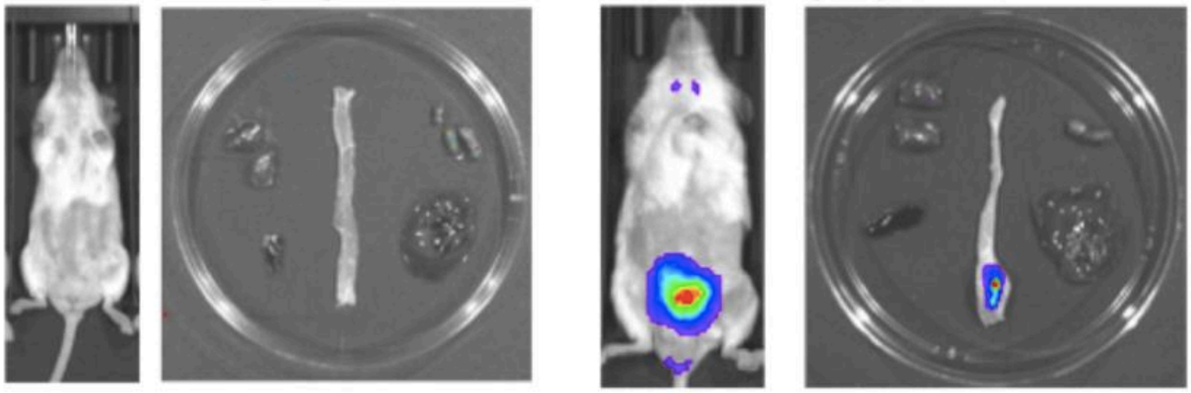

Figure 1. Using our mouse model of colorectal cancer, we administered luciferase tagged EcN to n=4 non-tumor bearing mice (left) and n=4 tumor-bearing mice (right). The EcN selectively colonised the tumors within tumor-bearing mice as shown by the luciferase (shown as colour in image) signal on the live animals. Following dissection, signal is localized to the tumour in the distal colon and not other nearby tissues (kidneys, spleen, liver), normal colon or stool.

3. AIM OF STUDY / RESEARCH QUESTIONS

To determine whether the probiotic EcN selectively colonizes colorectal neoplasia at both primary and metastatic sites in humans.

We expect to find that EcN is detectable within neoplastic tissue and thus establish the foundation for future translational research using this probiotic.

4. STUDY DESIGN

This study will be an interventional, double-blind, dual-centre, prospective clinical trial. Randomisation will not be used, as our research question is essentially whether EcN differentially colonises neoplastic tissue, in comparison to matched normal tissue from the same patient. The placebo group is included to use as a negative control cohort for our EcN detection assay, rather than to measure outcomes in the placebo vs. Mutaflor treated patient groups. As such we will recruit most trial participants to be in the Mutaflor study group, rather than the placebo group, to generate a larger number of samples with

which to answer the question of whether colonisation by EcN is higher in neoplastic compared to normal control tissue. Placebo patient samples negative for EcN are important controls for understanding the accuracy of the EcN assay and to set the limits of detection of the EcN assay.

Patients will be recruited from two sites, the Royal Adelaide Hospital (RAH) and St Andrew's Hospital. Tissue samples will be collected from consented patients during endoscopic procedures for polyp removal or surgery for bowel cancer and transported to the Gut Cancer Lab at SAHMRI, adjacent to the RAH. In the Lab, samples will be processed and cryostored and microbial contents of tissue homogenates analysed using PCR-based assays. Bowel cancer panel DNA sequencing of samples may also be undertaken in Adelaide (in the South Australian Genomics Consortium or ACRF Genomics Facility).

This project aims to work out whether a probiotic supplement can live specifically within colorectal precancers and cancers in humans. We do not believe that there are any alternative approaches to this study. We undertook a thorough literature review to check that this has not been previously published. We have already undertaken preliminary studies using mouse models of colorectal cancer. These experiments showed that oral administration of the probiotic EcN results in the specific growth of the probiotic bacteria in colorectal tumours and not normal, nearby colorectal tissue or other organs. The next critical step to determining whether this could be applied to detecting and combating colorectal cancer is working out whether this also occurs in humans. We need to use human subjects because we cannot mimic the complex route of bacterial uptake to growth in tumour tissue using isolated cells in the lab.

Pathologically surplus tissue from colorectal neoplasia and normal mucosa will be analysed, to test whether the EcN probiotic is present in neoplastic tissue at higher levels than in normal colonic mucosa, or in metastatic disease in comparison to normal resected liver. We will use a targeted PCR approach to detect EcN specifically in the samples, but may also undertake metagenomics sequencing to identify microbial species (the microbiome) more broadly present in each sample in the future. We can also determine EcN levels (if any) in participant blood samples as an additional control tissue.

5. STUDY SETTING/LOCATION

a. Sites

Patients will be recruited from two sites, the Royal Adelaide Hospital (RAH) and St Andrew's Hospital both in Adelaide, South Australia. Tissue samples were transported to the Gut Cancer Lab at the South Australian Health and Medical Research Institute (SAHMRI), adjacent to the RAH. In the Lab, samples will be processed and either stored or cultured and analysed. Colorectal cancer panel DNA sequencing of samples may also be undertaken in Adelaide (in the SAHMRI Genomics Facility or ACRF Genomics Facility).

6. STUDY POPULATION AND SETTING

Participants will be those scheduled for routine colonoscopy (RAH or St. Andrews) or diagnosed with CRC and referred to the CRC multidisciplinary team at the RAH or St Andrews for consideration of surgical resection of the tumour (and adjacent normal tissue). This MDT feeds into theatre lists with 2-5 CRC surgical resections each week at the RAH, with about 20 screening colonoscopies performed each week. The clinical team at the RAH and senior researchers from SAHMRI, all investigators on this

project, identified and approached potential study participants in clinic sessions at the RAH or St. Andrews >2 weeks before colonoscopy or surgery. We estimated that the 22 colon cancer and 44 colonoscopy participants could be recruited and take part in this study within 1 year, but the rectal cancer and liver resections being less frequent and so would require the full length, 5-year term requested for this study.

7. ELIGIBILITY CRITERIA

b. Inclusion criteria

Participants will be 18 years or older and undergoing endoscopic polyp removal or surgical resection of colorectal cancer by the colorectal team at the Royal Adelaide Hospital or St Andrews Hospital in more than 2 weeks from their recruitment date. Most participants will have confirmed cancer by pathology, but could also be included if pathology was inconclusive but a colorectal mass is being biopsied or resected. Previous treatment or radiation to the tumour site is permissible for this study.

c. Exclusion criteria

Participants must not be currently taking probiotics or antibiotics. The participant could be taking antibiotics immediately before commencing but not during probiotic dosing. This is similar to the dosing schedule in Suez et al, 2018[4].

8. STUDY OUTCOMES

a. Quantitative

i. Primary Outcome

The primary outcome of this study is to determine whether EcN probiotics administered to participants colonise neoplastic tissue more than nearby normal tissue.

ii. Secondary Outcome(s)

In the event probiotics do preferentially colonise neoplastic tissue, then the secondary outcomes will be to determine whether any other properties of the sample (eg. Tumour genetics, microbiome composition, clinical features) predict how well a lesion is colonised.

b. Qualitative

We estimate that this initial project will conclude within 5 years, and result in a determination of whether EcN preferentially colonises neoplastic over normal tissue in humans. Quantitative EcN-sequence specific PCR, the most sensitive method to detect EcN, will be used to measure probiotic levels in participant blood and tissue samples. Analysis of clinical information will be straight-forward, requiring only categorisation of samples according to tumour location and primary or metastatic status.

If successful, this will lead onto future applications in which we can engineer these bacteria to act as living reporters of neoplasia for CRC screening, prevention and potentially even therapy. At the time of completion of the project, biospecimens and data will be retained if a further ethically approved project takes the place of this study and should no further ethically approved project take the place of this study, then any unused tissue and clinical data will be destroyed.

Researchers will not feedback the results of research with the participants unless specifically contacted by participants using contact information provided on the participant information sheet. Data will be published in international, peer-reviewed journals and presented at national and international scientific conferences. All participant personal information will remain de-identified throughout the project and its dissemination and publication. The results of the research will guide the future plans of the project. If this probiotic does appear to have tumour homing abilities in humans, further ethics approval will be sought to validate any findings linking colonisation efficiency with properties of the neoplasia in a larger cohort and also to test new applications of genetically engineered forms of the probiotic.

9. STUDY PROCEDURES

a. Recruitment of participants

Study investigators will recruit consecutive eligible patients undergoing endoscopic polyp removal or surgical resection of colorectal cancer by the colorectal team at the Royal Adelaide Hospital or St Andrews Hospital in more than 2 weeks from their recruitment date. Potential participants will be approached by study investigators in clinic sessions at the RAH or St. Andrews >2 weeks before colonoscopy or surgery. It will be explained in a private setting that they were identified as a possible candidate for our research study because of their upcoming colonoscopy or surgery. A brief outline of our research group's background and the purpose of this project will be given. What is required of the participant, if they choose to participate, will then be clearly detailed as well as making them aware of their right to refuse to participate and that participation is voluntary. This information is all included on the participant information and consent form.

All participants in this study will be adults that are able to give informed consent. We will be obtaining written consent from these participants and all will have the opportunity to withdraw from the study at any time without affecting their medical care. During the consenting process the investigator will be individually responsible for making sure that each participant has given informed consent. This may be hindered by one of many factors, such as if the participants preferred language is not English. In this case the consenting researcher will ask for consent with a translator present. Similarly, the presence of a family member or friend will be encouraged to witness the consent and assist with communication if necessary. The witness will not be an investigator on the study. To ensure that the participant giving consent has understood what is involved with the project and what is required of them, before signatures are obtained specific points will again be addressed as detailed in the consent form and time will be given for questions to be thoroughly answered or clarified. If doubts still remain as to whether the participant fully understands the aims of the project and what their participation involves, they will be provided with the participant information sheet to take home and directed to the contact details of the principal investigator for any further questions they may have. All participants will be encouraged to contact the lead investigator, if they have questions during the study or would like to be contacted about outcomes of our research.

Participants will have been scheduled for routine colonoscopy (RAH or St. Andrews) or diagnosed with CRC and referred to the CRC multidisciplinary team at the RAH or St Andrews for consideration of surgical resection of the tumour (and adjacent normal tissue). This MDT feeds into theatre lists with 2-5 CRC surgical resections each week at the RAH, with about 20 screening colonoscopies performed each week. We estimate that the 22 colon cancer and 44 colonoscopy participants will be recruited and take part in this study within 1 year, but the rectal cancer and liver resections are less frequent and so will require the full length term requested for this study.

b. Study procedures

Participants will take either 2 tablets (10^9 CFU) per day of the probiotic EcN (Mutaflor) or placebo for 14-28 days, prior to surgery. Each participant will also contribute pathologically surplus tissue from colonoscopic biopsy prior to polypectomy or surgical resection, both cancer and normal adjacent tissue. They will also provide a 9ml blood sample at the time of their procedure. There will be no participant follow-up in addition to standard clinical care following their procedure, but participants are welcome to contact study investigators at any stage as outlined in the participant information sheet.

c. Methods of data collection

Tissue samples collected from study participants will be transported to the Gut Cancer Lab at the South Australian Health and Medical Research Institute (SAHMRI), adjacent to the RAH. In the Lab, samples will be processed and cryostored and microbial contents of tissue homogenates analysed using PCR-based assays by experienced molecular biologists.

Clinical information for each participant including DOB, sex, stage, histology, location of lesion, previous history of bowel cancer and previous treatment will be collected. Registrars undertaking Gastroenterology & Hepatology training at the RAH have been recruited to this project to access this clinical information. De-identified clinical information will be stored in an electronic database at SAHMRI. In the event of a participant withdrawing from the study all clinical information and tissue samples collected will be destroyed.

d. Access to Existing Data

**Name/Description of data**	**Royal Adelaide Hospital and St Andrews Hospital Medical Records**
**Data Custodian**	Royal Adelaide Hospital/CALHN and St Andrews Hospital
**Agency Type**	State
**Data Collection Format**	Re-identifiable
Variable	***Justification***
Patient_ID	To be able to link data to the correct patient we need to use the patient ID number for the patient for each hospital
Age at time of surgery	This information is relevant to understand if patient age may influence probiotic colonisation
Neoplasia Pathology report	This information is relevant to understand if type or location of neoplasia may influence probiotic colonisation
Treatment History	This information is relevant to understand if prior treatment may influence probiotic colonisation

e. Data Linkage Management

There is no data linkage for this project.

f. Safety considerations

This project uses the probiotic 'Mutaflor', containing *Escherichia coli* strain Nissle 1917 (EcN). It is manufactured by Herdeche (Germany) and supplied by Natural Therapy Imports (SA). Mutaflor is used for the relief and management of chronic constipation. The standard recommended dose for constipation is 2 Mutaflor capsules 3 times/day for the first 2 days and then from the third day onwards, 4 Mutaflor capsules per day. Mutaflor can be taken for up to 8 weeks at a time and is to be repeated periodically when necessary. For this study, dosage will be lower than the constipation dose at 2 tablets per day for 14-28 days. The dosing duration is variable because we do not yet know the optimal treatment window to enable colonisation of precancerous or cancerous lesions in humans. We have selected the 14-28 day window based on two previous studies; one in humans using a different probiotic formulation but using sophisticated analyses to determine the exact colonisation of sections of the gut and gut contents following probiotic administration, and the second is our previous work using the same probiotic (EcN) but in mice bearing colorectal tumours. The first is a recent clinical trial examining colonisation of the normal (not cancerous) human gastrointestinal tract by an 11-species containing probiotic[4]. Suez and co-workers treated participants for 4 weeks and then ascertained colonisation of various sections of the normal GIT mucosa using pinch biopsy samples similar to the samples that we will collect from colonoscopy. This study did not examine a shorter probiotic treatment window. Secondly we have shown in mice that 2-3 oral doses of EcN over a 1 week period is sufficient to promote highly specific colonisation of colorectal tumours over normal adjacent tissue. We would predict that colonisation of colorectal lesions by EcN will be more efficient in humans than the normal tissue and hence are interested in using both the 4-week treatment window used by Suez and co-workers, but also reducing the treatment duration to 2-weeks to see if we can still detect colonisation of lesions by EcN. The duration of EcN treatment pre-surgery will be considered when assessing outcomes of the study.

EcN is believed to act by modulating colonic motility via the production of short chain fatty acids. It is excreted by bowel movements. Mutaflor is in general very well tolerated. After the beginning of administration of Mutaflor to treat constipation at the higher dose, flatulence is common. Very rarely, undesired effects of the gastrointestinal tract such as abdominal pain, gut noises, loose stools or diarrhoea, nausea and vomiting may occur. Again very rarely, cases of skin reddening or rashes may occur. Also very rarely, cases of headache may occur. In the event of an adverse event, study doctors will be required to fill out the supplied adverse event report form, which will be sent to Dr Daniel Worthley (PI) and stored at SAHMRI. Dr Daniel Worthley will inform the HREC and RGOs of any adverse event that occurs. Mutaflor has no known contra-indications. Mutaflor has been provided for this study free-of-charge by the distributor. Mutaflor and placebo are stored at SAHMRI and will be directly supplied by study investigators to participants when they are recruited to the study in the pre-operative and pre-endoscopy RAH/St Andrews clinic sessions.

g. Data monitoring

Data monitoring will be the responsibility of the entire Investigator team. Investigators will use continuous vigilance to ensure data integrity, participant privacy and data confidentiality for potentially re-identifiable data and tracking and accountability for study documentation.

h. Protocol Deviations

A protocol deviation is any noncompliance with the study protocol, GCP, or HREC requirements.
The noncompliance may be either on the part of the participant, the investigator, or the study site staff. As a result of deviations, corrective actions are to be implemented promptly.

The principal investigator will use continuous vigilance to identify and report deviations within 72 hours of identification of the protocol deviation. All deviations must be addressed in study source documents, reported to the approving HREC(s) and site Research Governance Officer(s).

i. Unexpected or Serious Adverse Events

An unexpected adverse event is an unforeseen harmful, unpleasant or undesirable response, reaction or outcome experienced by a research participant or researcher. Such incidents may include unanticipated physical, psychological, emotional, cultural, financial or legal harm. It may also include where an unexpected event has occurred which may potentially harm participants, researchers, or the study organisation.

A serious adverse event is any untoward medical or psychological occurrence that results in death, is life threatening, requires inpatient hospitalisation or prolongation of existing hospitalization, or results in persistent of significant disability or incapacity.

The principal investigator will use continuous vigilance to identify and report adverse events within 72 hours of identification of the event to all approving HRECs and relevant Research Governance Officers.

10. DATA ANALYSIS

a. Quantitative

i. Sample size and statistical power

We aim to recruit

1. 22 consecutive patients diagnosed with primary colonic cancer (n=11 right-sided and n=11 left-sided)

2. 22 consecutive adult patients diagnosed with primary rectal cancer

3. 22 consecutive adult patients with colorectal cancer liver metastasis.

4. 44 consecutive patients with precancerous colonic lesions (n=22 right-sided and n=22 left-sided)

Patients in each group (1-4 above) will be assigned to probiotic treatment or to placebo.

This initial study is designed to determine whether there is any evidence of preferential colonisation of human neoplasia compared to normal adjacent tissue by the probiotic EcN. We expect a 10% drop-out rate of participants from this study eg. if the participant requires antibiotics during the trial, postponement of surgery or if participant decides to withdraw. As such we will recruit 22 participants for each group to enable collection from 20 participants/group. A group size of 20 (for groups 1-4 above), allowing up to 10 participants on placebo and a minimum of 10 on probiotic. These numbers were determined based on the experience of our microbiome team with a number of factors. These include participant to participant and gut location variability in probiotic colonisation efficiency (see [5]). The EcN probiotic colonisation efficiency of normal vs neoplastic human colon is not known (indeed this is why we are doing this study) and so is difficult to accurately estimate sample size required. From our mouse work using genetically identical animals on a controlled diet, there is a greater than 10,000-fold increase in probiotic bacteria levels in neoplastic tissue using sample sizes of 4-6 individuals.

ii. Statistical methods

Mean value of 4 technical replicates will be used per sample for the EcN specific and pan-microbial 16S quantitative PCR assays of patient tissue samples. Data will be displayed as a box plot depicting 25th-75th percentile and median per group, Wilcoxon test used to evaluate statistically significant differences in assay signal between matched normal and tumour tissue samples.

b. Qualitative

N/A

c. De-identification

Each participant will be allocated a participant number upon consenting so that any identifying information is removed from the collected biospecimens. This participant number will be used in any notes or publications arising from the research in order to protect the participant's identity.

Signed consent forms collected for this study will be kept and stored in paper format and also scanned electronically as a backup. All stored paperwork (consent forms containing participants name, hospital patient URN) will be filed and kept in a lockable filing cabinet in the principal investigators office area. The remaining information relevant to the participants sample or clinical information will be kept in a de-identified manner electronically in a database.
Identifiers will be removed from participant samples upon their receipt in the SAHMRI laboratory. The identifiers will be replaced with a de-identified code number. The electronic database, which will hold de-identified information (i.e. will contain participant's clinical information but de-identified by use of a code number) will be saved to a SAHMRI hard drive and accessible only by Drs Worthley, Woods, Wright and Vrbanac (named investigators). These investigators will be responsible for maintaining the accuracy and confidentiality of the database. They will disclose de-identified clinical information about the samples to other named investigators on this study.

11. DATA HANDLING AND RECORD KEEPING

a. Data Collection and Management Responsibilities

The data collection for this study involves: storage and cataloguing of signed consent forms and patient biosamples, and also an electronic database of de-identified patient information and SAHMRI research lab test results.
Consent forms collected for this study will be kept and stored in paper format and also scanned electronically as a backup. All stored paperwork (consent forms containing participants name, hospital patient URN) will be filed and kept in a lockable filing cabinet in the principal investigators office area. The remaining information relevant to the participants sample or clinical information will be kept in a de-identified manner electronically in a database.
The electronic database, which will hold de-identified information (i.e. will contain participant's clinical information but de-identified by use of a code number) will be saved to a SAHMRI hard drive and accessible only by Drs Worthley, Woods, Wright and Vrbanac (named investigators). These investigators will be responsible for maintaining the accuracy and confidentiality of the database. They will disclose de-identified clinical information about the samples to other named investigators on this study, but will undertake all data analysis and manuscript preparation at SAHMRI.

b. Study Records Retention

Stored tissue samples and associated (de-identified) clinical information will be kept for a period of up to 15 years due to SA Health legislative requirements. After the 15years, if no further ethically approved project takes place of this study, all data, biospecimens and clinical information will be destroyed.

De-identified biospecimens will be securely stored at SAHMRI and may be accessed by other researchers provided their research is ethically approved by an appropriately constituted HREC.

At the completion of this project, if no further ethically approved project takes place of this study, deidentified, electronic information and biosamples will be destroyed, stored paperwork will be disposed of in confidential document disposal.

12. PUBLICATION & INTELLECTUAL PROPERTY

Data will be published in international, peer-reviewed journals and presented at national and international scientific conferences.

a. Dissemination of results to participants

Outcomes of this project will not be directly disseminated to participants unless they have requested further contact from Study Investigators to share project outcomes. This is an initial study that aims to investigate whether probiotics that have been administered to humans for over 100 years, grow in and on cancers in humans, because it is those probiotics that may have the greatest value for delivering future therapies. There is no direct benefit to the participant or their families. All participants will be encouraged to contact the lead investigator, if they would like to be contacted about outcomes of our research during the consenting procedure and as detailed in our patient information sheet.

13. ETHICAL CONSIDERATIONS

a. Indemnity & Compensation for Injury

The University of Adelaide is indemnifying the study.

b. Vulnerable populations

All participants in this study will be adults that are able to give informed consent. We will be obtaining written consent from these participants and all will have the opportunity to withdraw from the study at any time without affecting their medical care. During the consenting process the investigator will be individually responsible for making sure that each participant has given informed consent. This may be hindered by one of many factors, such as if the participants preferred language is not English. In this case the consenting researcher will ask for consent with a translator present. Similarly, the presence of a family member or friend will be encouraged to witness the consent and assist with communication if necessary. The witness will not be an investigator on the study. To ensure that the participant giving consent has understood what is involved with the project and what is required of them, before signatures are obtained specific points will again be addressed as detailed in the consent form and time will be given for questions to be thoroughly answered or clarified. If doubts still remain as to whether the participant fully understands the aims of the project and what their participation involves, they will be provided with the participant information sheet to take home and directed to the contact details of the principal investigator for any further questions they may have. All participants will be encouraged to contact the lead investigator, if they have questions during the study or would like to be contacted about outcomes of our research.

c. Waiver of Consent

N/A, no waiver of consent will be sought.

d. Confidentiality

We will be collecting potentially re-identifiable health information from participants.

Signed consent forms collected for this study will be kept and stored in paper format and also scanned electronically as a backup. All stored paperwork (consent forms containing participants name, patient URN) will be filed and kept in a lockable filing cabinet in the principal investigators office area-but this means that patient samples are potentially re-identifiable.
Identifiers will be removed from participant samples upon their receipt in the SAHMRI laboratory. The identifiers will be replaced with a de-identified code number. The electronic database, which will hold de-identified information (i.e. will contain participant's clinical information but de-identified by use of a code number) will be saved to a SAHMRI hard drive and accessible only by Drs Worthley, Woods, Wright and Vrbanac (named investigators). These investigators will be responsible for maintaining the accuracy and confidentiality of the database.

e. Ethical Review

The study will be conducted in full conformance with principles of the "Declaration of Helsinki", Good Clinical Practice (GCP), the National Statement on Ethical Conduct in Human Research (NHMRC, 2007), Australian Code for the Responsible Conduct of Research (2007) and within the laws and regulations Australia.

Ethical approval has been obtained from the following HRECs:

- Central Adelaide Local Health Network Human Research Ethics Committee
- St Andrew's Hospital Human Research & Ethics Committee

f. Site/Governance Review

In accordance with the *SA Health Research Governance Policy Directive,* Site Specific Assessment (SSA) Approval has been sought from individual public health and research sites where the project is being conducted, including:

- University of Adelaide
- St Andrew's
- Royal Adelaide Hospital
- South Australian Health and Medical Research Institute

14. OUTCOMES AND SIGNIFICANCE

This clinical trial aims to evaluate the colonisation of matched human normal and neoplastic bowel tissue by the probiotic *E. coli* Nissle 1917 (EcN). If we can show that the probiotic EcN is enriched within neoplastic tissue in patients, as it is in mouse models of cancer, this will establish the foundation for future translational research using this probiotic for the detection and prevention of cancer.

15. REFERENCES

1. Danino T, Prindle A, Kwong GA, Skalak M, Li H, Allen K, Hasty J, Bhatia SN. Programmable

probiotics for detection of cancer in urine. Sci Transl Med. 2015 May 27;7(289):289ra84. doi: 10.1126/scitranslmed.aaa3519.

2. Hwang L, Fein SH, Ng K, D'Cruz O, Qazi S, Wang W, Nam D, Fong A. RNAi-mediated betacatenin knockdown in the gastrointestinal mucosa (GI) of patients with familial adenomatous polyposis (FAP): Results of START-FAP trial. Journal of Clinical Oncology 2017 35:15_suppl, e15065-e15065.

3. Petersen AM, Mirsepasi H, Halkjær SI, Mortensen EM, Nordgaard-Lassen I, Krogfelt KA.
Ciprofloxacin and probiotic Escherichia coli Nissle add-on treatment in active ulcerative colitis: a double-blind randomized placebo controlled clinical trial. J Crohns Colitis. 2014 Nov;8(11): 1498-505. doi: 10.1016/j.crohns.2014.06.001. Epub 2014 Jun 25. PMID: 24972748

4. Suez J, Zmora N, Zilberman-Schapira G, Mor U, Dori-Bachash M, Bashiardes S, Zur M, Regev-Lehavi D, Ben-Zeev Brik R, Federici S, Horn M, Cohen Y, Moor A, Zeevi D, Korem T, Kotler E, Harmelin A, Itzkovitz S, Maharshak N, Shibolet O, Pevsner-Fischer M, Shapiro H, Sharon I, Halpern Z, Segal E, Elinav E. Post-Antibiotic Gut Mucosal Microbiome Reconstitution Is Impaired by Probiotics and Improved by Autologous FMT. Cell 2018 174:1406-1423.

5. Zmora N, Zilberman-Schapira G, Suez J, Mor U, Dori-Bachash M, Bashiardes S, Kotler E, Zur M, Regev-Lehavi D, Brik RB, Federici S, Cohen Y, Linevsky R, Rothschild D, Moor AE, Ben-Moshe S, Harmelin A, Itzkovitz S, Maharshak N, Shibolet O, Shapiro H, Pevsner-Fischer M, Sharon I, Halpern Z, Segal E, Elinav E. Personalized Gut Mucosal Colonization Resistance to Empiric Probiotics Is Associated with Unique Host and Microbiome Features. Cell 2018 174:1388-1405.
