# Supplementary material for: Engineering tumor-colonizing E. coli Nissle 1917 for detection and treatment of colorectal neoplasia
Source: Nat Commun. 2024 Jan 20;15:646. doi: 10.1038/s41467-024-44776-4 (PMC10799955; doi:10.1038/s41467-024-44776-4)
Supplement: Supplementary file 3 — Reporting Summary [file 41467_2024_44776_MOESM3_ESM.pdf]

nature portfolio

Corresponding author(s): Susan Woods and Tal Danino

Last updated by author(s): November 5, 2023

Reporting Summary

Nature Portfolio wishes to improve the reproducibility of the work that we publish. This form provides structure for consistency and transparency in reporting. For further information on Nature Portfolio policies, see our Editorial Policies and the Editorial Policy Checklist.

Statistics

For all statistical analyses, confirm that the following items are present in the figure legend, table legend, main text, or Methods section.

n/a	Confirmed	
☐	☒	The exact sample size (*n*) for each experimental group/condition, given as a discrete number and unit of measurement
☐	☒	A statement on whether measurements were taken from distinct samples or whether the same sample was measured repeatedly
☐	☒	The statistical test(s) used AND whether they are one- or two-sided<br>*Only common tests should be described solely by name; describe more complex techniques in the Methods section.*
☒	☐	A description of all covariates tested
☐	☒	A description of any assumptions or corrections, such as tests of normality and adjustment for multiple comparisons
☐	☒	A full description of the statistical parameters including central tendency (e.g. means) or other basic estimates (e.g. regression coefficient) AND variation (e.g. standard deviation) or associated estimates of uncertainty (e.g. confidence intervals)
☐	☒	For null hypothesis testing, the test statistic (e.g. *F*, *t*, *r*) with confidence intervals, effect sizes, degrees of freedom and *P* value noted<br>*Give P values as exact values whenever suitable.*
☒	☐	For Bayesian analysis, information on the choice of priors and Markov chain Monte Carlo settings
☒	☐	For hierarchical and complex designs, identification of the appropriate level for tests and full reporting of outcomes
☒	☐	Estimates of effect sizes (e.g. Cohen's *d*, Pearson's *r*), indicating how they were calculated

Our web collection on statistics for biologists contains articles on many of the points above.

Software and code

Policy information about availability of computer code

Data collection	N/A
Data analysis	Graphpad Prism V.8 were used for general statistical analysis, FIJI was used for all image analysis

For manuscripts utilizing custom algorithms or software that are central to the research but not yet described in published literature, software must be made available to editors and reviewers. We strongly encourage code deposition in a community repository (e.g. GitHub). See the Nature Portfolio guidelines for submitting code & software for further information.

Data

Policy information about availability of data

All manuscripts must include a data availability statement. This statement should provide the following information, where applicable:

- Accession codes, unique identifiers, or web links for publicly available datasets
- A description of any restrictions on data availability
- For clinical datasets or third party data, please ensure that the statement adheres to our policy

All data and clinical trial study protocol is available in the main text or the supplementary materials.

Research involving human participants, their data, or biological material

Policy information about studies with human participants or human data. See also policy information about sex, gender (identity/presentation), and sexual orientation and race, ethnicity and racism.

Reporting on sex and gender	Male and female participants were 18 years or older
Reporting on race, ethnicity, or other socially relevant groupings	*Please specify the socially constructed or socially relevant categorization variable(s) used in your manuscript and explain why they were used. Please note that such variables should not be used as proxies for other socially constructed/relevant variables (for example, race or ethnicity should not be used as a proxy for socioeconomic status).* *Provide clear definitions of the relevant terms used, how they were provided (by the participants/respondents, the researchers, or third parties), and the method(s) used to classify people into the different categories (e.g. self-report, census or administrative data, social media data, etc.)* *Please provide details about how you controlled for confounding variables in your analyses.*
Population characteristics	Participants were those scheduled for routine colonoscopy (RAH or St. Andrews) or diagnosed with CRC and referred to the CRC multidisciplinary team at the RAH or St Andrews for consideration of surgical resection of the tumour (and adjacent normal tissue). This MDT feeds into theatre lists with 2-5 CRC surgical resections each week at the RAH. The clinical team at the RAH and senior researchers from SAHMRI, all investigators on this project, identified and approached potential study participants in clinic sessions at the RAH or St. Andrews >2 weeks before surgery. Table S1 in the manuscript describes the cohort characteristics, including sex. <br>Inclusion criteria: Male and female participants were 18 years or older and undergoing surgical resection of colorectal cancer by the colorectal team at the Royal Adelaide Hospital or St Andrews Hospital in more than 2 weeks from their recruitment date. Most participants have confirmed cancer by pathology, but were also be included if pathology was inconclusive but a colorectal mass is being resected. Previous treatment or radiation to the tumour site was permissible for this study. <br>Exclusion criteria: Participants must not be currently taking probiotics or antibiotics.
Recruitment	Adult participants undergoing surgical resection for primary colorectal cancer were recruited from St. Andrew's Hospital and Royal Adelaide Hospital, Adelaide (N=35). Written, informed consent was provided before participants were assigned to take either 2 tab-lets (10^9 CFU) per day of non-genetically modified EcN (Mutaflor) or placebo for 14 days, prior to their procedure. Patients and treating physicians were blind to active or placebo status. Surgical resection samples from normal and neoplastic tissue were collected from each participant at the time of their procedure. Participants were excluded if they took probiotics or antibiotics during the trial period.
Ethics oversight	The study was approved by the Human Research Ethics Committee of the Central Adelaide Lo-cal Health Network (HREC/18/CALHN/751) to meet the requirements of the National Statement on Ethical Conduct in Human Research. The study design and conduct complied with all relevant regulations regarding the use of human study participants and was conducted in accordance with the criteria set by the Declaration of Helsinki.

Note that full information on the approval of the study protocol must also be provided in the manuscript.

Field-specific reporting

Please select the one below that is the best fit for your research. If you are not sure, read the appropriate sections before making your selection.

[x] Life sciences [ ] Behavioural & social sciences [ ] Ecological, evolutionary & environmental sciences

For a reference copy of the document with all sections, see nature.com/documents/nr-reporting-summary-flat.pdf

Life sciences study design

All studies must disclose on these points even when the disclosure is negative.

Sample size	We calculate sample size in our studies by using a power analysis and appropriate statistical test in G*Power 3.1 software. Previous animal studies, or small pilot studies when necessary, served as the basis for calculations of expected averages and deviations used to calculate power, for which we set experiment studies to a value of 0.8. This typically results in an experimental group size to be n = 4–7, depending on the experiment. Sample size is explicitly stated for each experimental group for individual experiments in figure captions and data descriptions.
Data exclusions	No data excluded.
Replication	All in vitro experiments were successfully repeated at least once. In vivo efficacy experiments were repeated at least twice across multiple cohorts.
Randomization	Mice were randomized into groups prior to treatment with an approximately equal number of males and females in each group. <br>For human clinical trial, randomisation was not used, as our research question was whether EcN differentially colonises neoplastic tissue, in comparison to matched normal tissue from the same patient. The placebo group is included to use as a negative control cohort for our EcN

	detection assay, rather than to measure outcomes in the placebo vs. Mutaflor treated patient groups. As such we recruited most trial participants to be in the Mutaflor study group, rather than the placebo group, to generate a larger number of samples with which to answer the question of whether colonisation by EcN is higher in neoplastic compared to normal control tissue. Placebo patient samples negative for EcN were important controls for understanding the accuracy of the EcN assay and to set the limits of detection of the EcN assay.
Blinding	Animals were treated by a blinded independent researcher. For human trial, participants and treating physicians were blind to active or placebo status. Investigators were not blinded during data collection and analysis.

Reporting for specific materials, systems and methods

We require information from authors about some types of materials, experimental systems and methods used in many studies. Here, indicate whether each material, system or method listed is relevant to your study. If you are not sure if a list item applies to your research, read the appropriate section before selecting a response.

Materials & experimental systems

n/a	Involved in the study
☐	☐ Antibodies
☒	☐ Eukaryotic cell lines
☒	☐ Palaeontology and archaeology
☐	☒ Animals and other organisms
☐	☒ Clinical data
☒	☐ Dual use research of concern
☒	☐ Plants

Methods

n/a	Involved in the study
☒	☐ ChIP-seq
☒	☐ Flow cytometry
☒	☐ MRI-based neuroimaging

Antibodies

Antibodies used	HA-Tag C29F4 #3724 from Cell Signaling Technology; GranzymeB Leica Biosystems PA0291; CD3 Abcam 16669; CD8 catalog #CST98941 clone D4W2Z; Hypoxyprobe (1:200) Cat# HP12-200 Kit from Hypoxyprobe; RNA ISH staining, RNAscope 2.5 Detection Kit from Advanced Cell Diagnostics; Lipopolysaccharide (LPS, 1:500) Cat# HM6011-100UG from Hycult Biotech.
Validation	HA-Tag, GranzymeB, CD3 and CD8 antibodies were validated by Histowiz Inc.<br>Hypoxyprobe, LPS and RNAscope was validated and optimized using appropriate control tissues as follows. Hypoxyprobe; kidneys that contain naturally hypoxic regions from a Hypoxyprobe-treated mouse were utilized as the positive control, with the corresponding negative control of a CRC tissue sample from a mouse that had not received Hypoxyprobe treatment (Fig. S3). RNA ISH staining; the positive control tissue was a mouse CRC that was intratumourally injected with EcN-lux bacteria ex vivo (after tissue harvest but before sample embedding), negative control tissue was a mouse CRC sample without bacterial treatment and stained using the EcN-lux specific lux probe, and a CRC sample from a mouse treated with EcN-lux bacteria in vivo then stained with the negative control probe to unrelated gene DapB (Fig. S3). LPS staining was optimized on positive control EcN-treated mammary carcinoma and orthotopic CRC tumours, in comparison to PBS-treated negative control tissues (data not included).

Animals and other research organisms

Policy information about studies involving animals; ARRIVE guidelines recommended for reporting animal research, and Sex and Gender in Research

Laboratory animals	Mice were maintained in the South Australia Health and Medical Research Institute (SAHMRI) Bioresources facility in accordance with JAX USA animal husbandry protocols. Animals were given food and water ad libitum and housed in temperature- (kept between 19°C - 24°C with an average temperature of 21°C -22 °C), moisture-(humidity is kept between 45-55%), and light-controlled (12h light/ dark cycle) individually ventilated cage systems.<br><br>NOD.Cg-PrkdcscidIl2rgtm1Wjl/SzJ (NSG) mice (male and female,6–12 weeks old), C57BL/6 mice (male,10–12 weeks old) Wild-type littermates of APC-min and APC-min mice on the C57BL/6 background (male and female, ~12-15 week old)
Wild animals	No wild animals were used in this study.
Reporting on sex	Both males and female APC-min and NSG mice were used and no difference in colonization or therapeutic efficacy was observed between sexes. Only C57BL/6 male mice were used for orthotopic CRC model (Fig 2G-I), as the transplanted tumor organoids reject in female recipients, the original donor from which the organoids were generated was a male mouse - hence only male mice are used for this model.
Field-collected samples	No field collected samples were used in the study.

Ethics oversight	All animal experimentation related to the orthotopic CRC model was approved by the institutional animal ethics committee [South Australia Health and Medical Research Institute (SAHMRI) SAM-319, SAM-20-031]; All animal experimentation related to the ApcMin/+ mouse model of CRC was approved by the Institutional Animal Care and Use Committee (Columbia University, protocols AC-AAAN8002 and AC-AAAZ4470).

Note that full information on the approval of the study protocol must also be provided in the manuscript.

Clinical data

Policy information about clinical studies

All manuscripts should comply with the ICMJEguidelines for publication of clinical research and a completedCONSORT checklist must be included with all submissions.

Clinical trial registration	WHO Universal Trial Number U1111-1225-7729
Study protocol	Full protocol entitled "Colonisation of Probiotic in Neoplasia" was included with this submission.
Data collection	Tissue samples collected from study participants in surgical theatres at the Royal Adelaide Hospital and St. Andrew's Hospital, Adelaide, were transported to the Gut Cancer Lab at the South Australian Health and Medical Research Institute (SAHMRI), adjacent to the RAH. In the Lab, samples were processed and cryostored and microbial contents of tissue homogenates analysed using PCR-based assays by experienced molecular biologists. First participant recruited 7th March 2019, last participant 24th September 2019. Clinical information for each participant including DOB, sex, stage, histology, location of lesion, previous history of bowel cancer and previous treatment was collected, final follow-up of clinical records September 2023. Registrars undertaking Gastroenterology & Hepatology training at the RAH have been recruited to this project to access this clinical information. De-identified clinical information was stored in an electronic database at SAHMRI. In the event of a participant withdrawing from the study all clinical information and tissue samples collected was destroyed.
Outcomes	The primary outcome of this study was to determine whether EcN probiotics administered to participants colonise neoplastic tissue more than nearby normal tissue. This followed on from our observations in mouse models of CRC following oral probiotic administration. In the event probiotics did preferentially colonise neoplastic tissue, then the secondary outcomes were going to be to determine whether any other properties of the sample (eg. Tumour genetics, microbiome composition, clinical features) predict how well a lesion is colonised. The secondary outcomes (eg. microbiome analyses associated with colonization status) was discontinued due to smaller than expected sample size.<br>Quantitative EcN-sequence specific PCR, the most sensitive method to detect EcN, was used to measure probiotic levels in participant blood and tissue samples. This primary outcome of our study is reported in the manuscript.<br>Analysis of clinical information required only categorisation of samples according to tumour location and primary or metastatic status. This information is included in Supp Table 1, however due to smaller than expected sample size we were unable to perform any analysis of correlations between this clinical data and colonisation status of the lesions.
